# Impaired receptivity and decidualization in DHEA-induced PCOS mice

**DOI:** 10.1038/srep38134

**Published:** 2016-12-07

**Authors:** Shu-Yun Li, Zhuo Song, Min-Jie Song, Jia-Wen Qin, Meng-Long Zhao, Zeng-Ming Yang

**Affiliations:** 1 College of Veterinary Medicine, South China Agricultural University, Guangzhou, China

## Abstract

Polycystic ovary syndrome (PCOS), a complex endocrine disorder, is a leading cause of female infertility. An obvious reason for infertility in PCOS women is anovulation. However, success rate with high quality embryos selected by assisted reproduction techniques in PCOS patients still remain low with a high rate of early clinical pregnancy loss, suggesting a problem in uterine receptivity. Using a dehydroepiandrosterone-induced mouse model of PCOS, some potential causes of decreased fertility in PCOS patients were explored. In our study, ovulation problem also causes sterility in PCOS mice. After blastocysts from normal mice are transferred into uterine lumen of pseudopregnant PCOS mice, the rate of embryo implantation was reduced. In PCOS mouse uteri, the implantation-related genes are also dysregulated. Additionally, artificial decidualization is severely impaired in PCOS mice. The serum estrogen level is significantly higher in PCOS mice than vehicle control. The high level of estrogen and potentially impaired LIF-STAT3 pathway may lead to embryo implantation failure in PCOS mice. Although there are many studies about effects of PCOS on endometrium, both embryo transfer and artificial decidualization are applied to exclude the effects from ovulation and embryos in our study.

Polycystic ovary syndrome (PCOS), the most common endocrine disorder in women of reproductive age with an estimated prevalence of 5–10%, is one of the most common causes of female infertility^1^,^2^. What’s more, PCOS is the most common cause of anovulatory infertility and menstrual cycle abnormalities^3^. Women suffering from PCOS are characterized by hyperandrogenism and chronic anovulation. PCOS also increases the clinical risk of pregnancy complications compared with controls^2^.

The window of implantation is a limited time for blastocyst acceptance in the midsecretory phase of the menstrual cycle, and a state characterized by low androgen levels^4^. In addition, the endometrium in PCOS patients overexpresses androgen receptor and fails to downregulate estrogen receptor α in the window of implantation^5^. Because women with PCOS are anovulatory or oligo-ovulatory, there are suboptimal regulation by estrogen and suboptimal or absent progesterone, having an increased risk for the development of endometrial hyperplasia and cancer^6^,^7^.

Although ovarian dysfunction is an obvious cause of infertility in PCOS patients^8^, infertility caused by ovarian dysfunction can be treated with ovulation induction agents. Even after ovulation is pharmacologically restored, anovulatory patients have reduced cumulative pregnancy rates, and exhibit a higher rate of implantation failure and spontaneous miscarriage^9^. Even if the excellent embryos are selected for transfer, the successful rate in PCOS patients remain low^10^. PCOS is associated higher rates of early clinical pregnancy loss (30–50%)^11^. Prior studies indicate that HOXA-10, HOXA-11 and insulin-like growth factor binding protein 1 (IGFBP1) are decreased during the secretory phase in patients with PCOS^5^,^12^,^13^. Therefore, anovulation is not the only cause of infertility. Endometrial environment may be associated with low fertility in PCOS women.

A key determinant of adequate endometrial receptivity to embryo implantation is the level of estrogen^14^. Lactoferrin (Ltf) is an estrogen-responsive gene^15^. Previous studies show that some genes are critical for implantation in mice. On day 4 of pregnancy, Indian hedgehog (Ihh), an essential mediator of PGR action in the uterus, and critical in mediating the communication between the uterine epithelium and stroma required for embryo implantation^16^, is dynamically expressed at high levels in the luminal epithelium and endometrial glands^17^. SGK1, a kinase involved in epithelial ion transport and cell survival, is up-regulated in unexplained infertility, most prominently in the luminal epithelium^18^. MSX1 is strongly expressed in the uterine epithelium at the receptive phase and conditional deletion of uterine Msx1 causes impaired uterine receptivity^19^. Muc-1, an anti-attachment molecule, is down-regulated in the receptive stage^20^. Hand2 is a critical regulator of the uterine stromal-epithelial communication that directs proper steroid regulation conducive for the establishment of pregnancy^21^. Additionally, the phosphorylation and nuclear translocation of Stat3 in the luminal epithelium of mouse uterus is a good indicator of receptivity^22^.

Based on ethical consideration, it is impossible to analyze embryo implantation in PCOS patients. Many PCOS mouse models have developed, including dehydroepiandrosterone (DHEA)-induced, DHT-induced and letrozole-induced ones^23^,^24^,^25^. DHEA is one of the most abundant circulating androgens in PCOS patients^26^. DHEA-induced PCOS model has been established in different mouse strains (Parkes strain mice^27^, BALB/c mice^28^, C57BL/6 mice^23^ and FVB/NJ mice^29^).

Compared to human PCOS patients, DHEA-induced PCOS mouse model shares many of the salient features, such as hyperandrogenism, insulin resistance, altered steroidogenesis, abnormal maturation of ovarian follicles and anovulation^30^. Additionally, these DHEA-induced mice show infertility and more atretic follicles and follicular cysts in ovaries^31^,^32^,^33^. However, these DHEA-induced mouse models are mainly used for studying ovary^28^,^29^,^34^,^35^,^36^. Based on our knowledge, effects of PCOS on embryo implantation and decidualization in the DHEA-induced mice are still unknown. In our study, the rate of embryo implantation is obviously lower even if blastocysts from normal mice are transferred into PCOS mice. The expression of implantation-related genes is dysregulated in PCOS mice. The high level of estrogen and potentially impaired LIF-STAT3 pathway may lead to embryo implantation failure in PCOS mice.

## Materials and Methods

### Animal models and treatments

CD1 mice were used in this study and housed in a SPF animal facility with a controlled environment (22–24 °C and 60–70% relative humidity) and on a light/dark cycle (12 h light/12 h dark) with food and water ad libitum. Five female mice were housed per cage, and one male mouse per cage. All animal protocols were approved by the Animal Care and Use Committee of South China Agricultural University. All of the experiments were carried out in accordance with the approved guidelines by South China Agricultural University.

The mouse model of PCOS was developed by administering *Mus musculus* (strain CD1) with dehydroisoandrosterone (DHEA, LKT Laboratories) as described previously^37^,^38^. Briefly, female mice (25-day-old) were daily injected (*sub cutaneously*) with DHEA (6 mg/100 g body weight, 100 μl/mouse in sesame oil with 10% of 95% ethanol, Sigma) for 20 consecutive days. The vehicle control group was injected with 0.09 ml sesame oil and 0.01 ml 95% ethanol daily for 20 consecutive days.

DHEA-treated female mice were mated with normal fertile or vasectomized male CD1 mice in the afternoon to induce pregnancy or pseudopregnancy. The day when vaginal plug was detected was defined as day 1 of pregnancy. Pregnancy on days 1 and 4 was confirmed by recovering embryos from the oviducts and uteri. The implantation sites on day 5 were identified with intravenous injection of Chicago blue dye solution.

To induce artificial decidualization, 10 μl of sesame oil was injected into one uterine horn on day 4 of pseudopregnancy while the non-injected contralateral horn served as a control. Deciduoma was evaluated on day 8 of pseudopregnancy. Eight control mice and twelve PCOS mice were used in this treatment.

### Embryo collection

Blastocysts were flushed from uterine horns of mice at 9:00 on day 4 of pregnancy with M2 medium (Sigma). Zygotes were collected from oviducts of mice at 9:00 on day 1.

### Embryo transfer

Blastocysts were flushed with M2 medium from uterine horns of normal mice at 9:00 on day 4 of pregnancy, and transferred into control or DHEA-induced PCOS pseudopregnant recipients at 10:00 on day 4, respectively. All mice from vehicle control and PCOS groups were sacrificed 48 h after embryo transfer to visualize implantation sites. Ten control mice and eighteen PCOS mice were used in this treatment.

### Assay of serum 17β-estradiol

The orbital blood collected from six vehicle control mice and eight PCOS mice were rested for 30 min at 37 °C and spun at 1000 *g* for 15 min at 4 °C, respectively. The supernatants were collected and cryopreserved at −80 °C. Serum 17β-estradiol was determined using Mouse/Rat ELISA kits (11-Esthu-E01; Alpco, MA, USA). All samples were run in duplicate and diluted to fit within the standard curve.

### RNA extraction and real-time PCR

Tissue was collected and stored immediately at −70 °C. RNA was extracted using a standard TRI Reagent (Sigma)-based protocol, followed by quantification and quality assessment. Briefly, samples were added 500 ml TRI reagent and homogenized. Then the homogenate was centrifuged at 12,000 *g* for 10 minutes at 4 °C to remove the insoluble material. Samples were shook vigorously for 15 seconds, and stood for 2 minutes at room temperature. After a centrifugation at 12,000* g* for 15 minutes at 4 °C, the aqueous phase was transferred to a fresh tube and mixed with the same volume of 2-propanol. After the mixture was centrifuged at 12,000 *g* for 10 minutes at 4 °C, collected RNA pellet was digested with RQ1 deoxyribonuclease I (Promega, Fitchburg, WI). Then 5 μg of RNA was reverse-transcribed into cDNA with PrimeScript reverse transcriptase reagent kit (Perfect Real Time, TaKaRa). For real-time PCR, each reaction (20 μl) contained 50 ng cDNA,10 μl SYBR Premix Ex Taq (Takara) and 4 μmol of forward and reverse primers. The conditions used for real-time PCR were as follows: 95 °C for 10 s followed by 39 cycles of 95 °C for 5 s and 60 °C for 34 s. All reactions were run in triplicate. Rpl7 was used for normalizing the expression level of each gene. Data from real-time PCR were analyzed using the ΔΔCt method^39^. Primer pairs specific for each gene were designed using Primer preimer 5.0. Primers used for real-time PCR were listed in Table 1.

### *In situ* hybridization

RNA was isolated from mouse uteri on day 4 of pregnancy, reverse transcribed and amplified with the primers for mouse Ihh, Sgk1 and Msx1. The amplified fragment of each gene was cloned into pGEM-T (pGEM-T Vector System 1; Promega, Madison, WI) plasmid in accordance with the manufacturer instructions and verified by sequencing (Sangon Biotech, Shanghai). To prepare the templates, the plasmids were amplified with the primers for T7 and SP6. Digoxigenin-labeled antisense or sense cRNA probes were transcribed *in vitro* using a digoxigenin RNA labeling kit (Roche Applied Science). Primers used for *in situ* hybridization were listed in Table 1.

*In situ* hybridization was performed as previously described^40^. Frozen uterine sections of six vehicle control mice and eight PCOS mice (10 μm) were mounted onto 3-aminopropyltriethoxysilane (Sigma)-coated slides and fixed in 4% paraformaldehyde solution in PBS for 1 h. Hybridization was performed at 55 °C for 16 h. Then sections were incubated in anti-digoxigenin antibody conjugated to alkaline phosphatase (1:5000; Roche Applied Science). The positive signal was visualized with the buffer containing 0.4 mM 5-bromo-4-chloro-3-indolyl phosphate (Amresco) and 0.4 mM nitro blue tetrazolium (Amresco) as a dark brown color. All of the sections were counterstained with 1% methyl green. Digoxigenin-labeled sense probe for each gene was also hybridized and served as a negative control. Endogenous alkaline phosphatase activity was inhibited with 2 mM levamisole (Sigma). The whole images were performed for densitometry by ImageJ.

### Immunohistochemistry

Paraffin-embedded uterine sections of six vehicle control mice and eight PCOS mice (5 μm thick) were deparaffinized in xylene, rehydrated through a graded series of ethanol, and washed in water. Antigen retrieval was performed in 10 mM sodium citrate buffer (pH 6.0) by microwaving for 10 min and then cooling to room temperature. Endogenous horseradish peroxidase (HRP) activity was inhibited with 3% H_2_O_2_ for 15 min. After blocked with 10% horse serum at 37 °C for 1 h to prevent nonspecific binding, sections were incubated with rabbit anti-ER (1:800, Santa), rabbit anti-MUC-1 (1:400; Thermo), rabbit anti-PR (1:1200, Thermo), rabbit anti-p-Stat3 (1:400, Cell Signaling Technology), rabbit anti-Ki67 (1:400, Thermo) or goat anti-Hand2 (1:200, Santa) diluted in PBS at 4 °C overnight, respectively. Followed by washing and incubating with biotin-labeled rabbit anti-goat IgG antibodies or goat anti-rabbit IgG antibodies (Zhongshan Golden Bridge, Beijing, China) for 30 min, respectively, sections were incubated with streptavidin-HRP complex (Zhongshan Golden Bridge) for 30 min. The positive signals were visualized using DAB Horseradish Peroxidase Color Development Kit according to the manufacturer’s protocol (Zhongshan Golden Bridge) as a reddish-brown color. The sections were counterstained with hematoxylin. The whole images were performed densitometry by ImageJ.

### Statistical analysis

The number (N) of mice used was shown in each figure. The number (N) of samples used in each analysis was also shown in each figure. P value < 0.05 was considered statistically significant. All analyses were performed using the GraphPad Prism^®^ software (GraphPad Software Inc., San Diego, CA, USA).

## Results

### Fertility in DHEA-induced PCOS mice

**Figure 1:**
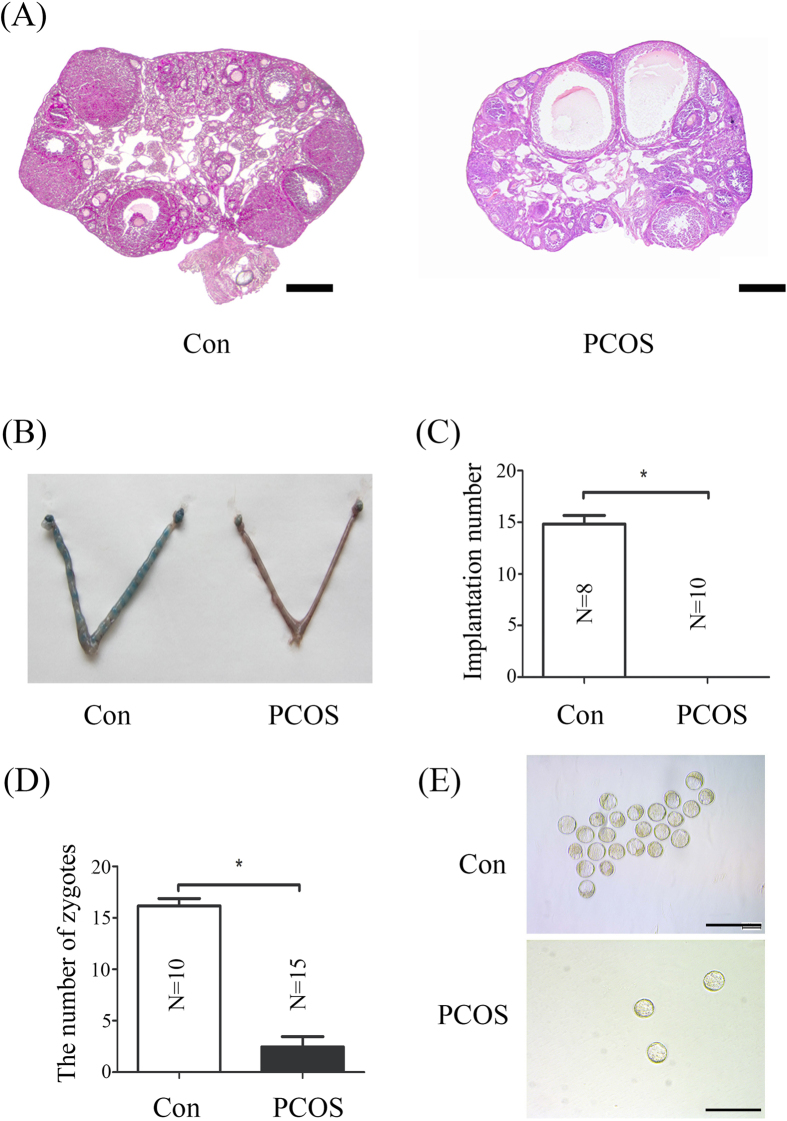
Fertility in DHEA-induced PCOS mice. (**A**) Hematoxylin and eosin stained-ovary sections from control and DHEA-induced PCOS mice. (**B**) A representative photograph showing mouse uteri on day 5 between vehicle control and PCOS mice. (**C**) The number of implantation sites per mouse in vehicle control group and PCOS group. (**D**) The number of zygotes collected from vehicle control group and PCOS group. (**E**) Morphology of blastocysts collected from vehicle control group and PCOS group on day 4. N, number of mice; *p < 0.05; Bar = 300 μm.

In vehicle control group ovary was normal and contained large numbers of follicles at various stages of development. PCOS ovaries exhibited multiple cystic follicles with a large fluid-filled antrum and degenerate granulosa cell layers (Fig. 1A).

To investigate whether embryo implantation occurs, vehicle control and PCOS mice were sacrificed on day 5 of pregnancy. Implantation sites were detected in vehicle control group, and the number of implantation sites was at a normal range (~15 sites). However, no implantation site was detected in PCOS group, suggesting complete pregnancy failure (Fig. 1B and C). Then we examined if embryos normally develop in PCOS mice. Although the number of blastocysts collected from PCOS mice was much less, the morphology of blastocysts from PCOS mice was normal and similar to vehicle control mice. We also examined the number of zygotes collected from PCOS mice. The number of zygotes from PCOS mice was reduced.

### Embryo implantation in PCOS mice

**Figure 2:**
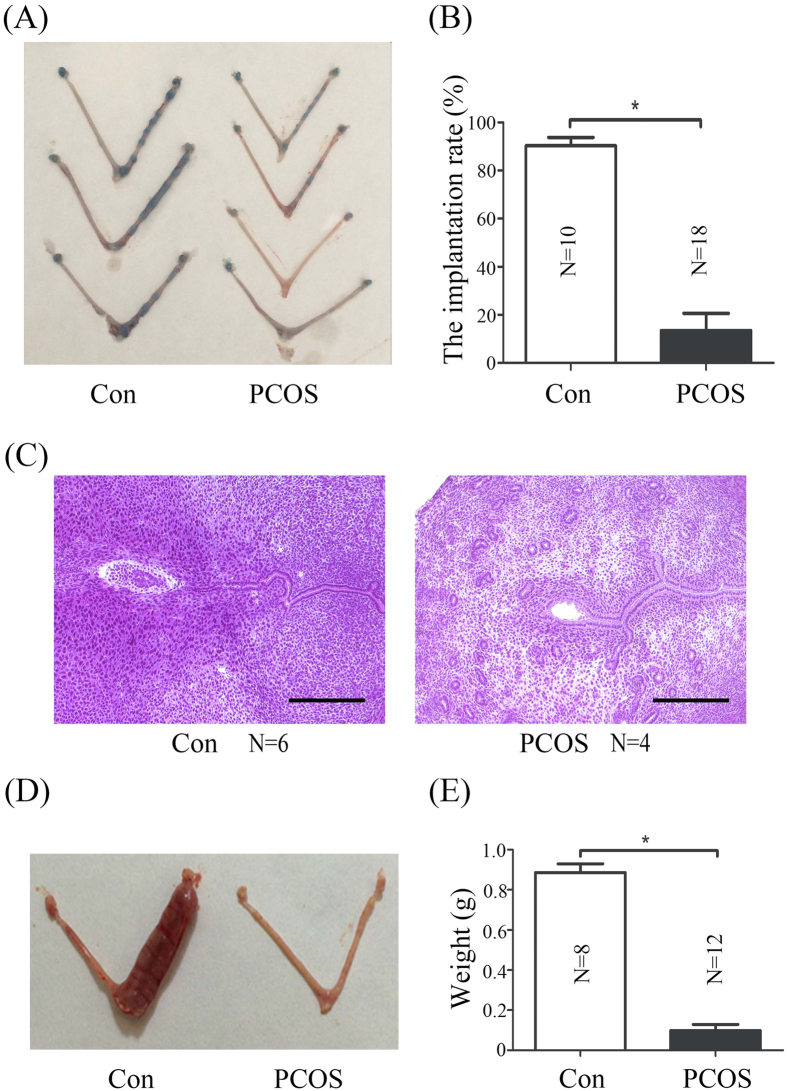
Embryo implantation and decidualization in PCOS mice. (**A**) Uteri on day 6 of pseudopregnancy after blastocysts collected from normal mice were transferred into vehicle control and PCOS pseudopregnant recipients on day 4, respectively. All mice were sacrificed 48 h after transplantation. (**B**) The implantation rate after blastocysts collected from normal mice was transferred into vehicle control and PCOS pseudopregnant recipients on day 4, respectively. (**C**) Hematoxylin-Eosin-stained uterine sections from vehicle control and PCOS mice on day 6 (48 h after transplantation). (**D**) Deciduoma on day 8 of pseudopregnancy after artificial decidualization is induced in vehicle control and PCOS mice. (**E**) The weights of deciduoma in vehicle control and PCOS mice. N, number of mice; *p < 0.05; Bar = 300 μm.

To exclude the possibility that implantation failure in PCOS mice is from the low number of blastocysts, blastocysts recovered from normal mice were transferred into vehicle control and PCOS pseudopregnant recipients, respectively. The implantation rate of vehicle control group was higher than that in PCOS group (Fig. 2A and B). Moreover implanted embryos of PCOS mice were smaller (Fig. 2C). These results indicated that uterine environment may play a significant role in PCOS-linked infertility. The notion was supported by the finding that the decidua of PCOS induced mice was smaller and lighter (Fig. 2D and E), suggestive of impaired decidualization.

### Expression of implantation related genes and proteins in PCOS mice

**Figure 3:**
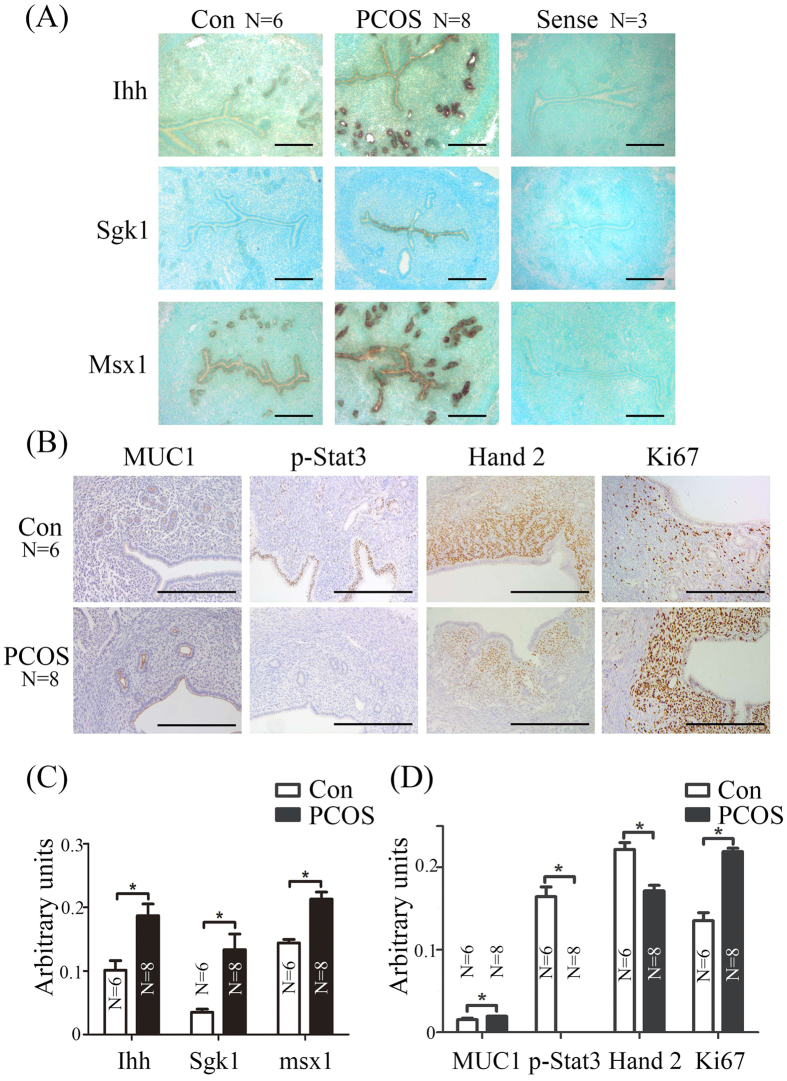
Expression of implantation-related genes in PCOS mice on day 4. (**A**) *In situ* hybridization showing the expression of Ihh, Sgk1 and Msx1 in mouse uteri from vehicle control and PCOS mice on day 4. (**B**) Immunohistochemical staining showing the expression of MUC1, p-Stat3, Hand 2 and Ki67 in mouse uteri from vehicle control and PCOS mice on day 4. Quantitative analysis of *In situ* hybridization (**C**) and immunohistochemical staining (**D**) using ImageJ. N, number of sections; *p < 0.05; Bar = 300 μm.

In PCOS mice, Ihh was strongly expressed in the glandular epithelium (Fig. 3A and C). Strikingly, *in situ* hybridization analysis of uterine sections revealed Sgk1 markedly increased in luminal epithelium of PCOS mice (Fig. 3A and C). The expression of Msx1 in luminal epithelium and glandular epithelium in vehicle control group was weaker than PCOS group. In PCOS mice, Muc1 was detected in the luminal epithelium and glandular epithelium in vehicle control mice (Fig. 3B and D). The signals of p-Stat3 were mainly observed in luminal epithelium, and weakly in glandular epithelium and stromal cells in vehicle control mice, and were hardly observed in PCOS mice (Fig. 3B and D). In addition, Hand2 expression was weaker and Ki67 was stronger in PCOS group (Fig. 3B and D).

### High level of estrogen disrupts LIF-STAT3 signal pathway in PCOS Mice

**Figure 4:**
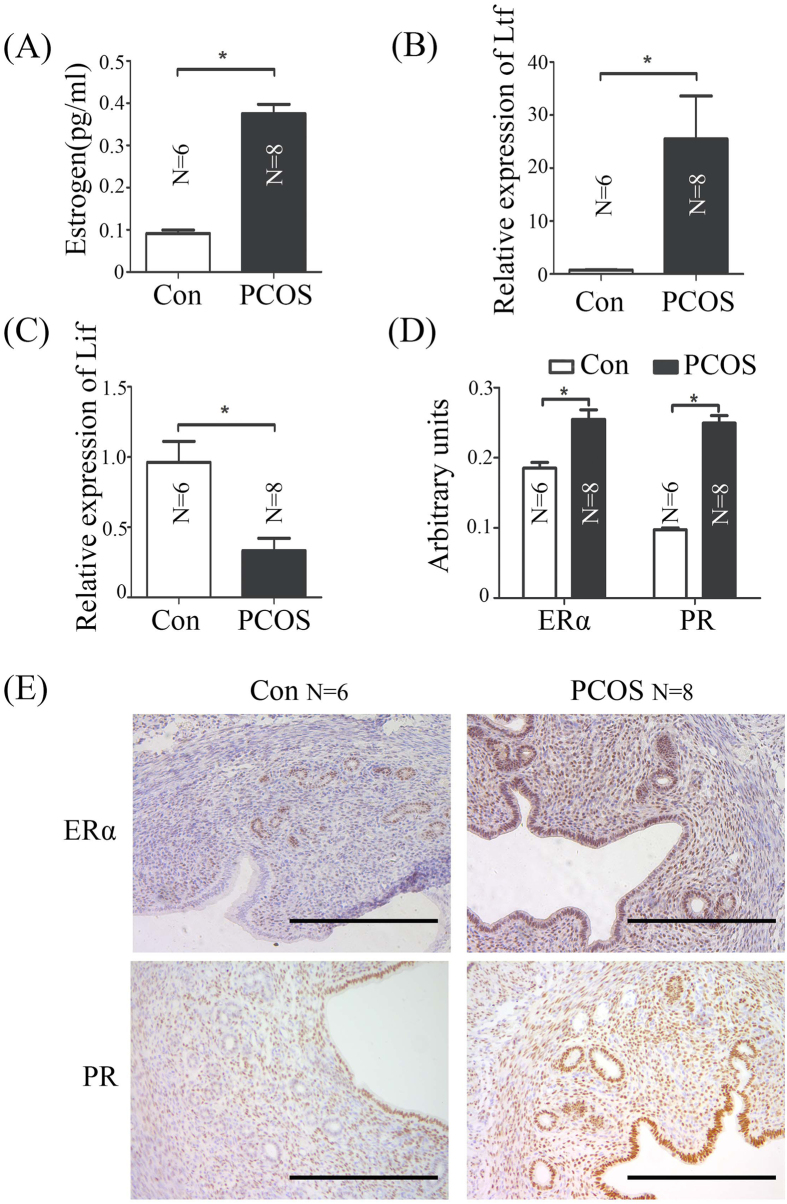
Estrogen-related changes in vehicle control and PCOS mouse uteri on day 4. (**A**) Serum estradiol levels in vehicle control and PCOS mice. (**B**) Real-time RT-PCR of Ltf mRNA level in the uterus from vehicle control and PCOS mice on day 4. (**C**) Real-time RT-PCR of Lif mRNA level in the uterus from vehicle control and PCOS mice on day 4. (**D**) Quantitative analysis of immunohistochemical staining using ImageJ. (**E**) Immunohistochemical staining showing the expression of ERα and PR proteins in mouse uteri from vehicle control and PCOS mice on day 4. N, number of mice; *p < 0.05; Bar = 300 μm.

In our study, the level of estrogen is more than 4 fold higher than vehicle control mice (Fig. 4A). Ltf was significantly up-regulated in PCOS group compared to vehicle controls (Fig. 4B). The expression of Lif is regulated by estrogen was quantified in uterus from control and PCOS mice by real-time RT-PCR (Fig. 4C and D). Our data indicated that the super-physiological level of estrogen might be a reason for infertility in PCOS mice by disruption LIF-STAT3 signal pathway. Estrogen receptor (ERα) and progesterone receptor (PR) were up-regulated in PCOS mice by immunohistochemistry (Fig. 4D and E).

## Discussion

This study has induced PCOS in mice using DHEA and show that uterine receptivity is impaired. These mice also exhibit high circulating estrogen and altered expression of implantation-related genes. Uterine decidualization is also compromised in PCOS mice. Although there are many studies about effects of PCOS on endometrium, our study used models of embryo transfer and artificial decidualization to exclude the effects from ovulation and embryos.

Anovulatory infertility is a common cause among reproductive-aged women with PCOS^3^. Our data also indicate that the number of zygotes from PCOS mice is significantly reduced (Fig. 1D). Although ovarian dysfunction is pharmacologically restored by ovulation induction agents^8^, PCOS patients still exhibit a higher rate of implantation failure and spontaneous miscarriage^9^. These studies suggest that the successful rate in PCOS patients remain low even if the excellent embryos are selected for transfer^10^. We also showed that blastocysts from normal mice are unable to implant in PCOS recipients (Fig. 2). Additionally, morphologically normal blastocysts from PCOS mice were not implanted (Fig. 1). Our data also indicated that implantation-related genes are dysregulated in PCOS mouse uterus, suggesting uterine environment in PCOS mice is altered.

Uterine sensitivity to implantation is divided into three principal phases: pre-receptive (days 1–3), receptive (day 4) and non-receptive (refractory; day 5 onward) in mice^41^,^42^. Progesterone and estrogen are the precondition for uterine transition into the receptive phase^14^,^43^. Estrogen is a critical determinant for specifying the duration of the window of uterine receptivity. A high level of estrogen will shorten implantation window^14^. In this study, endogenous estrogen level in PCOS mice is more than 4 fold higher than vehicle control mice, which is confirmed by the increase of Ltf, an estrogen-responsive gene (Fig. 4A and B). DHEA is the major source for estrogen formation in the fetoplacental unit during pregnancy^44^. In DHEA-treated pregnant mice, the excess of androgen causes a decrease of serum progesterone level and an increase of serum estradiol level in early pregnant mice^45^. Additionally, women with PCOS are anovulatory or oligo-ovulatory, and their endometrium have suboptimal regulation by estrogen, and suboptimal or absent progesterone^6^,^7^,^46^.

Lif is strongly expressed in the receptive uterus and stimulated by estrogen^47^,^48^. Lif deficiency will lead to implantation failure^49^. However, Lif expression is down-regulated in PCOS mouse uterus (Fig. 4B). Additionally, Lif concentration is significantly lower in the follicular fluid of PCOS patients compared with controls^50^. The down-regulation of Lif in PCOS mice may be due to complex endocrine disorder. Stat3 can be phosphorylated and nuclear translocated by Lif^22^. Conditional uterine deletion of Stat3 impairs embryo implantation^51^,^52^,^53^,^54^. Our data suggest that implantation failure in PCOS mice may be caused by dysregulated LIF-STAT3 pathway.

In our study, ovulation problem is not only the reason for infertility in PCOS mice. The abnormal expression of uterine receptivity-related genes may contribute to the low pregnancy rate observed in PCOS. Although estrogen level is crucial in regulating the window of uterine receptivity in mice^14^, ovarian hyperstimulation does not adversely affect uterine receptivity for implantation in IVF/ET programs^55^, meaning that the range of estrogen level is less restrictive in humans than in mice. Nonetheless, understanding molecular signaling networks that coordinate strategies for successful implantation and decidualization in PCOS may lead to approaches for improving the outcome of pregnancy in PCOS patients.

## Additional Information

**How to cite this article**: Li, S.-Y. *et al*. Impaired receptivity and decidualization in DHEA-induced PCOS mice. *Sci. Rep.* **6**, 38134; doi: 10.1038/srep38134 (2016).

**Publisher's note:** Springer Nature remains neutral with regard to jurisdictional claims in published maps and institutional affiliations.

## Figures and Tables

**Table 1:** Primers used in this study.

Gene	Primer sequences	Size	Application
*Msx1*	5-CACTTCCTCCTGGTTGTCG-3 5-TGGGCTCCTTGCTTTCT-3	331 bp	*In Situ* Hybridization
*Sgk1*	5-GAACACGGCTGAGATGTA-3 5-TAATACGACTCACTATAGGG-3	484 bp	*In Situ* Hybridization
*Ihh*	5-CTGCGGTTCTGTCTGTTCCT-3 5-CCAGCAGTCCATACTTATTTCG-3	472 bp	*In Situ* Hybridization
*Ltf*	5-AGCCAACAAATGTGCCTCTTC-3 5-CCTCAAATACCGTGCTTCCTC-3	119 bp	Real-time PCR
*Lif*	5-AAAAGCTATGTGCGCCTAACA-3 5-GTATGCGACCATCCGATACAG-3	98 bp	Real-time PCR
T7	5-TGTAATACGACTCACTATAGGG-3		*In Situ* Hybridization
SP6	5-CTATTTAGGTGACACTATAGAAT-3		*In Situ* Hybridization
